# Case Report: Cerebral Nocardiosis Caused by *Nocardia cyriacigeorgica* Detected by Metagenomics in an Apparently Immunocompetent Patient

**DOI:** 10.3389/fimmu.2022.719124

**Published:** 2022-02-03

**Authors:** Virginie Courbin, Quentin Riller, Jean-Louis Amegnizin, Guillaume Gricourt, Vanessa Demontant, Vincent Fihman, Cecile Angebault, Matthieu Mahevas, Géraldine Gaube, Laëtitia Coutte, Jean-Michel Pawlotsky, Raphaël Lepeule, Christophe Rodriguez, Paul-Louis Woerther

**Affiliations:** ^1^ Department of Microbiology, AP-HP, Henri Mondor University Hospital, Université Paris-Est, Créteil, France; ^2^ Department of Radiology, AP-HP, Henri Mondor University Hospital, Université Paris-Est, Créteil, France; ^3^ NGS Plateform, IMRB Institute, AP-HP, Henri Mondor University Hospital, Université Paris-Est, Créteil, France; ^4^ EA Dynamyc 7380, Université Paris Est Créteil-Ecole nationale Vétérinaire de Maisons-Alfort, Créteil, France; ^5^ Internal Medicine Department, AP-HP, Henri Mondor University Hospital, Université Paris-Est, Créteil, France

**Keywords:** meningoencephalitis, *Nocardia*, metagenomic, molecular biology, infection

## Abstract

We report a case of meningoencephalitis due to *Nocardia cyriacigeorgica* diagnosed with metagenomics, while all the standard methods were negative. This diagnosis made adaptation of antimicrobial treatment possible and led to the discovery of a rare, acquired immunodeficiency syndrome.

## Introduction

Cerebral nocardiosis is a rare and serious infection, occurring mainly in immunocompromised patients, that presents most commonly as brain abscesses (1–3). Because this infection is not associated with pathognomonic symptoms, its diagnosis is often difficult and delayed, resulting in a mortality rate of 57% (2). Metagenomics is a method based on next-generation sequencing, which allows the detection of pathogens without *a-priori*, whatever their nature (bacteria, viruses, fungi). In addition to pathogen identification, metagenomics can also provide quantification and genomes analysis (4). Here, we report the first case of *Nocardia cyriacigeorgica* meningoencephalitis diagnosed by clinical metagenomics.

## Case Presentation

A 66-year-old man, with no notable past medical history, came to the emergency department in April 2017, complaining about flu-like symptoms, including headache, asthenia and fever. Symptoms began 10 days before, and persisted despite a short co-amoxiclav self-medication. The biology report showed hyponatremia (129 mmol/L), with a mild biological inflammatory syndrome (C-reactive protein (CRP): 24.1 mg/L; leukocytes: 11500/mm^3^ including 88% polymorphonuclear cells). No altered consciousness or symptoms of meningitis were noticed. The clinical diagnosis of influenza was retained and a symptomatic treatment was prescribed. The following day, the patient came back with neurological impairment (somnolence, stupor, inability to respond to doctor’s requests) and fever at 38.3°C. His condition quickly deteriorated and he was admitted with febrile coma without focal neurological sign in the intensive care unit (ICU) for mechanical ventilation. Empirical treatment combining cefotaxime, amoxicillin, gentamicin and acyclovir was initiated.

**Figure 1:**
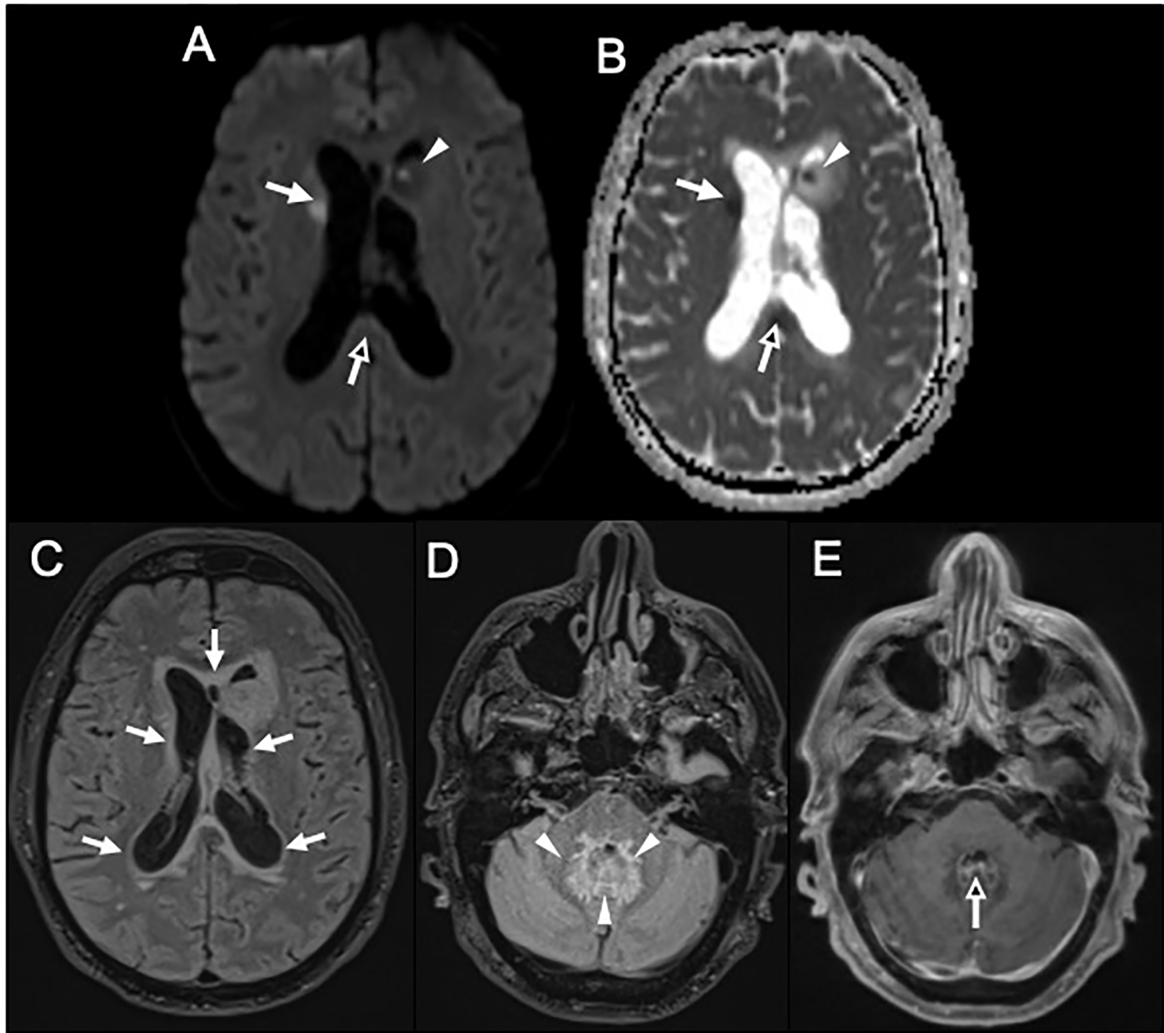
**(A, B)** Magnetic resonance imaging; **(A)** (Axial Diffusion Tensor Imaging (DTI) 6 directions 3,5mm slice thickness) and **(B)** [apparent diffusion coefficient (ADC)] panels show 9 mm acute ischemic lesion of the right caudate nucleus (**A**, **B**, white arrows), cytotoxic lesions of corpus callosum splenium multifactorial (**A**, **B** black arrows) and a 10 mm abscess of left caudate with peripheral edema (**A**, **B**, head arrows). **(C–E)** Magnetic resonance imaging; **(C, D)** (co-registration of 3D FLAIR 1mm slice thickness) and E (post-contrast three-dimensional T1-weighted turbo spin echo (3D T1-w TSE) 1mm slice thickness) panels show a T2FLAIR hyperintensity halo around lateral ventricles without enhancement, related to transependymal edema secondary to acute hydrocephalus (**C**, white arrows). A thick halo of T2FLAIR hyperintensity around the fourth ventricle (**D**, white head arrows) associated to a thin enhancement of the ependymal lining of the fourth ventricle (**E**, black arrow) were also evidenced in relation with an cerebral arteritis secondary to the meningoencephalitis.

**Figure 2:**
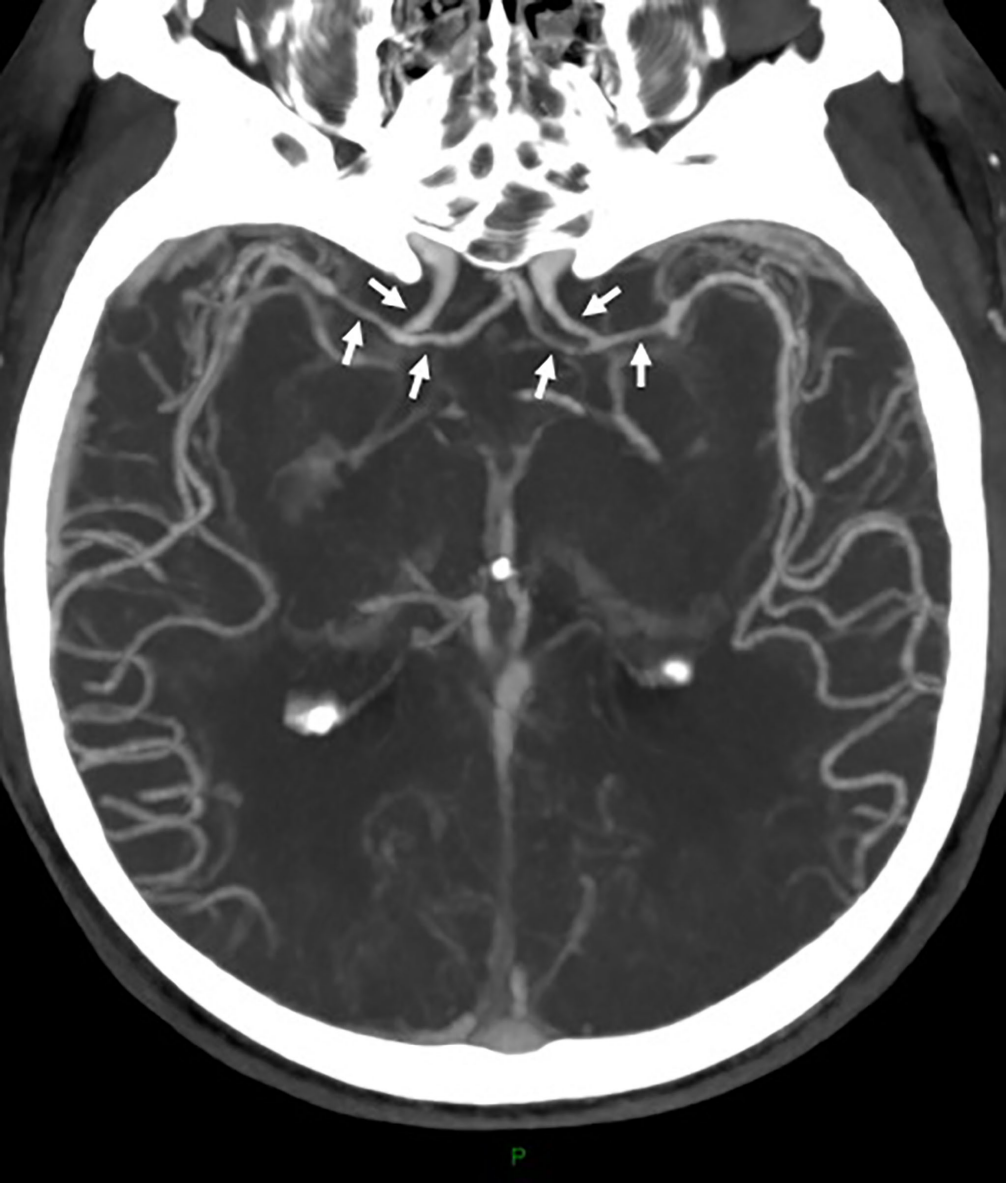
CT-Scan angiography shows discreet and bilateral stenoses of the intracranial internal carotid arteries and of the anterior cerebral arteries (A1 and M1 segments) (arrows).

The first cerebrospinal fluid (CSF) showed meningitis with 750 leukocytes/mm^3^ (76% polymorphonuclear cells), hyperproteinorachia (2.2 g/L) and hypoglycorachia (1.3 mmol/L, with 7.9 mmol/L glycaemia). Conventional microbiological investigations including standard, mycobacterial and fungal cultures, a commercial PCR system for detecting 16S rDNA (Molzym GmbH & Co. KG, Germany) and an in-house multiplex PCR (including *Listeria monocytogenes, Streptococcus pneumoniae and Neisseria meningitidis*) were negative. Human immunodeficiency virus (HIV) serology and first-line autoimmune disorder exploration (including plasmatic protein electrophoresis and lymphocytes phenotyping) were also negative. In this context of possible pyogen abscess or cerebral tuberculosis, metronidazole and anti-tuberculosis treatment (isoniazid, rifampin, pyrazinamide and ethambutol) were empirically added at day 1. On day 2, a second CSF was sampled. Decreased element counts were observed (50 leukocytes/mm^3^, 52% of polymorphonuclear cells), but standard and mycobacterial examinations remained negative. Magnetic resonance imaging showed a 10 mm abscess of the left caudate nucleus with surrounding edema associated with ependymitis of the fourth ventricle, acute hydrocephalous with transependymal edema, a 9-mm acute ischemic lesion of the right caudate nucleus with multifocal cerebral arteries irregularities and cytotoxic lesions of corpus callosum splenium (**Figure 1**). CT-Scan angiography performed at day 4 confirmed multifocal intra-cranial arteries stenoses, corresponding to cerebral arteritis secondary to the meningoencephalitis (**Figure 2**). In addition to antibiotics, dexamethasone (10 mg x4/day) was initiated and pursued with three pulsed intravenous methylprednisolone (1 g) relayed by oral corticosteroid therapy (1 mg/kg). On day 10, the third CSF showed 1130 leukocytes/mm^3^ (82% of polymorphonuclear cells). Conventional microbiological explorations (as previously cited) were again negative. Despite the absence of known immunosuppression, *Nocardia* specific culture was conducted (culture media BCYE was seeded and incubated at 37°C in aerobic atmosphere during two weeks) and remained negative. From day 11, in the absence of any microbiological documentation, only anti-tuberculosis treatment and oral corticosteroids were continued whereas other empirical antibiotics were stopped. Neurological condition improved and the patient was transferred to the Internal Medicine department with persistent cognitive impairment.

To further investigate a possible infectious etiology, the third CSF sample was submitted in our laboratory to clinical metagenomics, which is used in our hospital as a last-resort approach in patient with high suspicion of infection but negative microbiological analysis. A specific extraction combining mechanical (bead biting), enzymatical (proteinase K based) and chemical (lysis buffer) steps was performed before DNA and RNA library prep using Nextera and Total RNA kits (Illumina), and sequencing with NextSeq500 (Illumina) (4, 5). Bioinformatic analysis was performed using the MetaMIC software (4, 5). From a total of 36’882’731 reads generated from the CSF sample, 7’862 matched with *Nocardia cyriacigeorgica*’s genome, corresponding to a 2.13x10^-4^ pathogen/human nucleic acid ratio and a concentration of 250 genome copies/mL. This sample was also sent to the French Observatory of Nocardiosis (Lyon), where a specific PCR confirmed the presence of *Nocardia*, as well as species identification. Finally, the diagnosis of cerebral nocardiosis due to *N. cyriacigeorgica* with central nervous system vasculitis was retained. In the absence of possible phenotypic susceptibility testing, resistance genes were sought from metagenomics data. After alignment of raw reads with BWA (Burrows-Wheeler Aligner) on the reference genome identified by MetaMIC, we found a consistent coverage on overall the genome (**Supplementary Figure 1**, GenBank PRJNA640158). However, although the coverage almost encompassed the complete genome of the reference strain, the sequencing depth was insufficient to allow the formal identification of resistance genes, probably because of the early initiation of antibiotic treatment. There are currently no treatment recommendations for cerebral nocardiosis. However, in cerebral or disseminated nocardiosis, a combination of antibiotics is most often used, although no comparative studies have been carried out. The antibiotics most frequently used in the literature are cotrimoxazole, amikacin and imipenem (or broad-spectrum cephalosporins) (6). In absence of specific recommendations on cerebral nocardiosis treatment and because *N. cyriacigeorgica* belongs to drug resistance pattern type VI characterized by resistance to penicillin and susceptibility to broad-spectrum cephalosporins, imipenem, sulfonamide, amikacin and linezolid (1), administration of imipenem-cilastatin associated with trimethoprim-sulfamethoxazole was started, leading to a rapid improvement of the patient’s neurological condition, apyrexia and a decrease of the inflammatory syndrome.

Three weeks after hospital admission, the clinical evolution was marked by a febrile respiratory distress episode that was ascribed to putative invasive pulmonary aspergillosis, according to the *Asp*ICU criteria in critically ill patients (7). The patient had a compatible lung CT-scan while it was normal at admission, as well as several microbiological criteria: direct examination showing branching hyphae and culture positive with *Aspergillus* section Fumigati in bronchoalveolar lavage (BAL), positive seric and BAL galactomannan antigens and positive *Aspergillus* spp. PCR in pleural fluid. Specific culture and PCR of *Nocardia* on bronchial aspiration sample were negative. Amphotericin B was initiated and then replaced by voriconazole for a total length of six weeks, followed by posaconazole prophylaxis. The succession of two invasive infections suggestive of a deficiency in neutrophil phagocytic function (8) led to a more complete exploration of the patient’s immunity. The presence of anti-granulocyte-macrophage colony-stimulating factor (GM-CSF) antibodies (55 UI/L) resulted in diagnosis of anti-GM-CSF antibody-mediated acquired immunodeficiency. The patient was successfully treated with anti-CD20 (rituximab 375 mg/m², 2 courses) and concomitant GM-CSF replacement was initiated with sargramostim (a recombinant GM-CSF that functions as an immune-stimulator) and stopped six months later. Two years later, the patient was considered cured under trimethoprim-sulfamethoxazole prophylaxis.

## Discussion

*Nocardia* spp. are environmental Gram-positive rods responsible for rare and severe opportunistic infections in humans. Because antibiotic susceptibility profiles differ according to the species, precise identification is important in *Nocardia* infections (1). For this patient, the absence of bacterial detection by 16S rDNA was probably the consequence of an insufficient extraction yield associated with that method, leading to a false negative result. Indeed, the extraction method used for the metagenomics analysis in our laboratory has received special attention during its development steps, combining both mechanic, enzymatic and chemical lysis.

In our case, clinical metagenomics was capable to identify *N. cyriacigeorgica*, one of the most frequent *Nocardia* involved in human diseases (9), as responsible for meningoencephalitis. Even though *Nocardia* species are saprophytic and ubiquitous bacteria that reside in soils, the hypothesis of contamination from an environmental source was ruled out, not only because *Nocardia* spp. are rarely (if ever) contaminants in bacteriology laboratories, but also because the environmental controls performed at the same time as the patient sample were negative for this bacterium. Although cerebral nocardiosis generally occurs in immunocompromised patients as a result of hematogenous dissemination from a primary site (10), no pulmonary or cutaneous portal of entry was evidenced in our case. However, the presence of anti-GM-CSF autoantibodies may explain the occurrence of this rare infection in this patient. Indeed, the association between anti-GM-CSF autoantibodies and infection with opportunistic pathogens such as *Nocardia*, *Cryptococcus* or *Histoplasma*, has been documented (11–13), in relation with a probable underlying phagocyte dysfunction (11). Nevertheless, the frequency of the association with aspergillosis remains to be determined, since the role of GM-CSF in the recruitment of phagocytes into the lung in response to *Aspergillus* infections could possibly play a role (14). This case illustrates the strong potential of clinical metagenomics to identify rare pathogens, even in the absence of any clinical context or without any knowledge of the state of immunodeficiency.

## Data Availability Statement

The datasets presented in this study can be found in online repositories. The names of the repository/repositories and accession number(s) can be found below: https://www.ncbi.nlm.nih.gov/genbank/, PRJNA640158.

## Ethics Statement

The study was approved by the Henri Mondor Hospital Institutional Review Board (IRB), Créteil, France (IRB number: 00011558; approval number: 2020-082). The patients/participants provided their written informed consent to participate in this study. Written informed consent was obtained from the [individual(s) AND/OR minor(s)’ legal guardian/next of kin] for the publication of any potentially identifiable images or data included in this article.

## Author Contributions

CR and P-LW designed and directed the project, supervised by J-MP. VD performed the experiments. GGr, J-LA, and VC analyzed the data. VC, QR, and P-LW wrote the manuscript. MM, LC, GGa, VF, CA, and RL contributed to the interpretation of the data. All authors reviewed the results and approved the final version of the manuscript.

## Funding

This work has been funded by academic institution.

## Conflict of Interest

The authors declare that the research was conducted in the absence of any commercial or financial relationships that could be construed as a potential conflict of interest.

## Publisher’s Note

All claims expressed in this article are solely those of the authors and do not necessarily represent those of their affiliated organizations, or those of the publisher, the editors and the reviewers. Any product that may be evaluated in this article, or claim that may be made by its manufacturer, is not guaranteed or endorsed by the publisher.

## Supplementary Material

Supplementary Figure 1 Coverage of the reference genome of *Nocardia cyriacigeorgica* after alignment of raw reads obtained by metagenomics. The 3.1Mb gap potentially corresponds to genes belonging to the accessory genome.
