# Supplementary figures and images for: Case Report: Cerebral Nocardiosis Caused by Nocardia cyriacigeorgica Detected by Metagenomics in an Apparently Immunocompetent Patient
Source: Front Immunol. 2022 Feb 3;13:719124. doi: 10.3389/fimmu.2022.719124 (PMC8852340; doi:10.3389/fimmu.2022.719124)

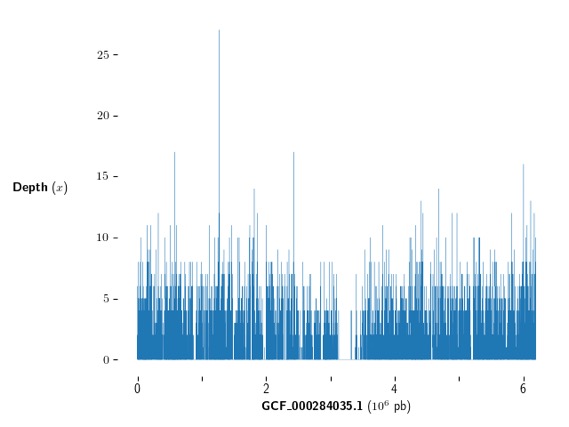

Supplement: Supplementary Figure 1 — Coverage of the reference genome of Nocardia cyriacigeorgica after alignment of raw reads obtained by metagenomics. The 3.1Mb gap potentially corresponds to genes belonging to the accessory genome. [file Image_1.jpeg]
